# Effect of bone cement distribution on adjacent disc degeneration after vertebral augmentation for osteoporotic vertebral compression fractures in aging patients

**DOI:** 10.3389/fsurg.2023.1256401

**Published:** 2023-09-01

**Authors:** Zhen Zhang, Jialang Zhang, Baorong He, Qi Dong, Dingjun Hao

**Affiliations:** Department of Spine Surgery, Xi'an Honghui Hospital, School of Medicine, Xi'an Jiaotong University, Xi'an, China

**Keywords:** cement distribution, disc degeneration, vertebral augmentation, osteoporotic vertebral compression fractures, vertebroplasty

## Abstract

**Background:**

The influence of vertebral augmentation on adjacent intervertebral discs remains controversial. The purpose of this study is to evaluate the effect of bone cement distribution on adjacent disc degeneration after vertebral augmentation for osteoporotic vertebral compression fractures (OVCFs).

**Methods:**

Patients with single level OVCF and upper endplate injury who underwent vertebral augmentation were enrolled. The patients were divided into four groups: Group A: bone cement contacted both the cranial and the distal endplates; Group B: bone cement only contacted the cranial endplate; Group C: bone cement only contacted the distal endplate; and Group D: bone cement contacted neither the cranial nor the distal endplates. The cranial discs of the fractured vertebrae were defined as adjacent discs and the upper discs proximally to the adjacent discs were defined as control discs. Degenerative disc change (DDC) was defined as a deteriorated postoperative Pfirrmann score compared with the preoperative score on MR images. The number of DDC cases and the disc heights were analyzed among the groups.

**Results:**

A total of 184 patients with an average follow-up time of 28.6 months were included. The number of DDC cases in the adjacent discs was significantly higher than in the control discs in groups A (*p* < 0.001), B (*p* = 0.002), and D (*p* = 0.028), whereas the difference in group C was not statistically significant (*p* = 0.237). The incidence of adjacent disc degeneration was significantly higher in group A than that in group C (*p* = 0.06). The adjacent disc heights decreased significantly in groups A, B, and D (*p* < 0.001, *p* < 0.001, and *p* = 0.012, respectively), but did not decrease significantly in group C (*p* = 0.079). However, no statistical differences were detected among the four groups with respect to the preoperative adjacent disc height, follow-up adjacent disc height, preoperative control disc height, or follow-up control disc height.

**Conclusion:**

Bone cement distribution influences adjacent disc degeneration after vertebral augmentation in OVCFs. Cement distribution proximal to the injured endplate can accelerate adjacent disc degeneration, and cement in contact with both the cranial and distal endplates can induce a higher incidence of adjacent disc degeneration.

## Introduction

Osteoporotic vertebral compression fractures (OVCFs), one of the most common and severe complications of osteoporosis, are becoming more common among the aging population. Vertebral augmentation was first applied by American doctors in patients with OVCFs in 1993 and obtained satisfactory efficacy. Since then, vertebral augmentation techniques, including percutaneous vertebroplasty and posterior kyphoplasty, have been widely used in the treatment of OVCFs and have received international recognition. Several studies show that vertebral augmentation improves clinical outcomes because it can achieve instant pain relief and stabilization of the fractured vertebral body (1–4). Nevertheless, several reports have indicated that vertebral augmentation may negatively affect adjacent segments (5, 6). Previous studies have mainly focused on the influence of vertebral augmentation on the adjacent vertebral body, as cement injection has been reported to increase the risk of adjacent vertebral fractures. Recently, the impact of vertebral augmentation on adjacent intervertebral discs, which has rarely been discussed, has been increasingly concerning. However, several studies that have attempted to determine whether vertebral augmentation can aggravate the degeneration of adjacent intervertebral discs have reached contradictory conclusions. Qian et al. (7) first conducted a prospective study and discovered a significantly higher incidence of adjacent disc degeneration above the fractured vertebrae in the cement augmentation group than in the control group. These findings were confirmed by retrospective studies by Lu et al. (8) and Wang et al. (9). However, a long-term follow-up study by König et al. (10), come to a different conclusion that vertebral cement augmentation through kyphoplasty had no significant influence on disc degeneration.

Cement distribution pattern is reportedly related to the occurrence of adjacent vertebrae fracture (11–14). These studies indicated that the cement distribution pattern may have an impact on the regional biomechanical stress. Moreover, cement distributed along the endplates can interfere with the endplate nutritional pathway and induce intervertebral disc degeneration in porcine and rabbit models (15, 16). Thus, the distribution of cement, which might have an impact on adjacent discs by altering both the regional biomechanical stress and the endplate nutritional pathway, should not be ignored when discussing the influence of vertebral augmentation on adjacent disc degeneration. Neglecting cement distribution patterns can lead to biased conclusions. However, to our knowledge, the influence of the cement distribution pattern on adjacent intervertebral discs has never been discussed. This study aimed to evaluate the effect of bone cement distribution on adjacent disc degeneration after vertebral augmentation in osteoporotic vertebral compression fractures.

## Materials and methods

### Study population

Clinical data and radiographic images of patients with OVCF who underwent vertebroplasty at our spine surgery center between January 2015 and December 2020 were retrospectively reviewed. As endplate injury has been reported to affect intervertebral disc degeneration (17), we only included patients with upper endplate injuries in this study to reduce bias. The inclusion criteria were as follows: (1) patients with low back pain who were diagnosed with a fresh single vertebral compression fracture of the lumbar spine (L1–L5) with upper endplate injury, and with no compression of the spinal cord or cauda equina; (2) age ≥55 years; (3) bone mineral density (BMD) of lumbar spine *T* score ≤−2.5; (4) Pfirrmann score of the adjacent intervertebral discs above the fractured vertebral body ≤2; (5) at least 2 years follow-up. The exclusion criteria were as follows: (1) vertebral pathological fracture caused by infection or tumor, (2) previous history of lumbar vertebral fracture or lumbar surgery, (3) cement leakage into intervertebral discs, (4) severe adjacent disc injury, and (5) cement concentrated on one side of the vertebral body on the frontal x-ray image.

### Operation procedure

All PVP procedures were performed using a unilateral transpedicular approach, with the patient in the prone position under local anesthesia. The fractured vertebrae were located, and a puncture needle was inserted through the unilateral pedicle paths under the guidance of C-arm fluoroscopy. The end of the needle was located in the middle of the vertebra on the anteroposterior film, and at 1/3 anterior-mid of the vertebral body on the lateral film. High-viscosity bone cement was slowly injected into the vertebral body using a hydraulic delivery system. After the injection was completed, the needle was removed, and the cement volume was recorded.

### Evaluation of clinical and radiological parameters

Age, sex, body mass index (BMI), number of smokers, and fracture segments were recorded. All patients were followed up for at least 2 years after surgery. Radiography, computed tomography, and MR imaging were performed before surgery. Radiographs were obtained on the first day, 3 months, 1 year, and 2 years after surgery, and MRI scans were performed only at the last follow-up visit for economical reasons.

**Figure 1:**
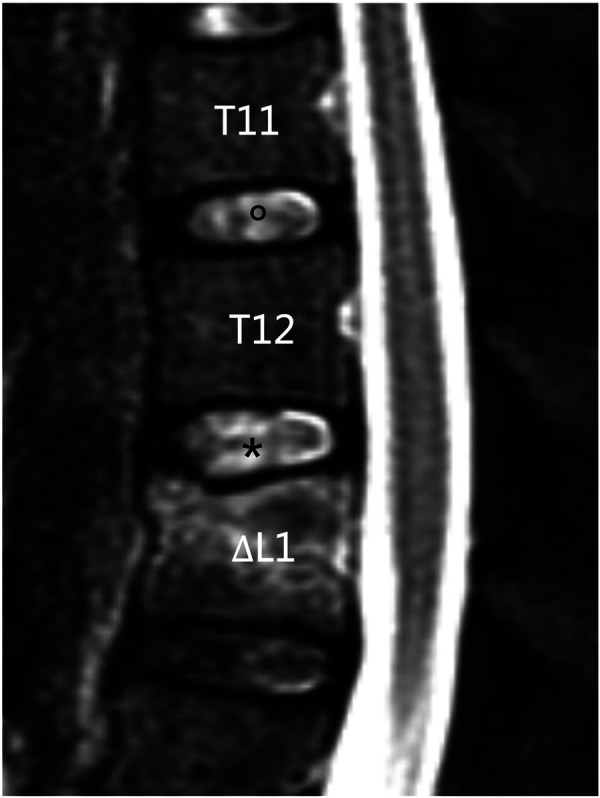
Definition of adjacent discs and control discs. In the mid-sagittal view of MRI, L1 was the fractured vertebral body, T12/L1 (*) disc was defined as the adjacent disc, and T11/T12 (∘) disc was defined as the control disc.

The cranial discs of the fractured vertebrae were defined as adjacent discs and the upper discs proximal to the adjacent discs were defined as control discs (Figure 1). The degree of disc degeneration was evaluated using the Pfirrmann scoring system (18). Degenerative disc change (DDC) was defined as a deteriorated postoperative Pfirrmann score compared with the preoperative score (excluding deterioration from grade 1 to grade 2). Disc height was defined as the mean of the anterior, middle, and posterior disc heights on midsagittal T1W MR, according to a previous report (8).

### Grouping method

**Figure 2:**
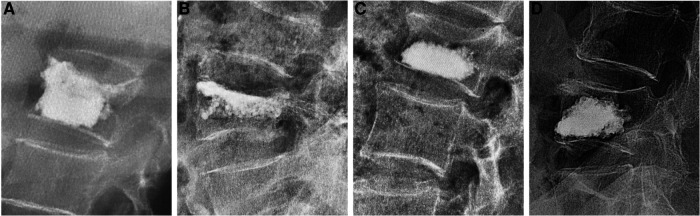
Four types of bone cement distribution: (**A**) bone cement contacted both the cranial and the distal endplates; (**B**) bone cement only contacted the cranial endplate; (**C**) bone cement only contacted the distal endplate; (**D**) bone cement contacted neither the cranial nor the distal endplate.

According to lateral radiographs taken on the first day after surgery, patients were divided into four groups. Group A: bone cement contacted both the cranial and the distal endplates; Group B: bone cement only contacted the cranial endplate; Group C: bone cement only contacted the distal endplate; and Group D: bone cement located in the middle of the vertebral body, contacting neither the cranial nor distal endplate. In this study, “contact” was defined as bone cement covering at least 50% of vertebral endplates on lateral x-ray images (Figure 2).

### Statistical analysis

Statistical analyses were performed using SPSS 26.0 (Chicago, Armonk, NY, USA). Continuous variables are presented as mean ± standard deviation (SD). Counting data are expressed as numbers. The Kruskal-Wallis test was used to analyze patient age, BMI, time of follow-up, bone cement volume, and disc height among the groups. Pearson's chi-square test or Fisher's exact test was used to compare patient sex, number of smokers, fracture segment, and number of DDCs. Differences were considered statistically significant at *p* < 0.05.

## Results

A total of 184 patients including 51males and 133 females were finally enrolled in this study. The mean follow-up period was 28.6 months (ranging from 24 to 49 months). There were 73 patients in group A, 62 patients in group B, 28 patients in group C, and 21 patients in group D. No significant differences were found among the groups with respect to age, sex ratio, BMI, number of smokers, fracture segment, or follow-up time (Table 1).

**Table 1:** Demographic and clinical characteristics of patients.

Characteristics	Group A (*n* = 73)	Group B (*n* = 62)	Group C (*n* = 28)	Group D (*n* = 21)	*p* value
Age	64.4 ± 6.2	66.0 ± 6.4	64.8 ± 6.4	64.9 ± 7.4	0.443
Gender (male/female)	20/53	17/45	10/18	4/17	0.640
BMI (kg/m^2^)	22.0 ± 4.2	21.5 ± 3.4	23.3 ± 2.5	21.1 ± 3.3	0.312
Smoker number	11	7	3	1	0.208
Fracture segment					0.881
L1	32	30	17	9	
L2	22	16	5	6	
L3	10	11	4	2	
L4	7	4	2	4	
L5	2	1	0	0	
Bone cement volume (ml)	4.8 ± 1.1	4.1 ± 1.3	4.6 ± 1.8	4.7 ± 1.5	0.195
Follow-up period (months)	30.3 ± 6.1	27.2 ± 5.2	26.1 ± 3.9	29.9 ± 5.0	0.387

At the final follow-up, 102 (55.4%) patients were found to have degeneration of the adjacent disc, of whom 48 (65.8%) were in group A, 32 (51.6%) in group B, 10 (35.7%) in group C, and 12 (57.1%) in group D. Forty-eight (26.1%) patients had control disc degeneration, of whom 22 (30.1%) were in group A, 15 (24.2%) in group B, 6 (21.4%) in group C, and 5 (23.8%) in group D. The number of DDC cases in the adjacent discs was significantly higher than in the control discs in groups A (*p* < 0.001), B (*p* = 0.002), and D (*p* = 0.028), whereas the difference in group C was not statistically significant (*p* = 0.237). The number of DDC cases in the control discs was not significantly different among the four groups (*p* = 0.775). However, the number of DDC cases in the adjacent discs, was significantly different among the four groups (*p* = 0.047). The number of DDC cases was significantly higher in group A than that in group C (*p* = 0.06). The difference in the DDC number in the adjacent discs between groups A and B (*p* = 0.096), groups A and D (*p* = 0.469), groups B and C (*p* = 0.162), groups B and D (*p* = 0.661), and groups C and D (*p* = 0.136) was not statistically significant (Table 2).

**Table 2:** Numbers of DDC cases in adjacent discs and control discs at follow-up.

		Group A	Group B	Group C	Group D	*p* value
Adjacent disc	DDC	48	32	10	12	0.047
	Non DDC	25	30	18	9	
Control disc	DDC	22	15	6	5	0.775
	Non DDC	51	47	22	17	
*p* value		<0.001	0.002	0.237	0.028	

The adjacent disc heights decreased significantly in groups A, B, and D (*p* < 0.001, *p* < 0.001, and *p* = 0.012, respectively), but did not decrease significantly in group C (*p* = 0.079). The follow-up control disc heights were not significantly different from the preoperative control disc heights in all of the four groups (*p* = 0.089, *p* = 0.138, *p* = 0.269, and *p* = 0.301, respectively). No statistical difference was detected among the four groups with respect to the preoperative adjacent disc height, follow-up adjacent disc height, preoperative control disc height, nor follow-up control disc height (*p* = 0.346, *p* = 0.519, *p* = 0.496, *p* = 0.218, respectively) (Table 3).

**Table 3:** Adjacent disc heights and control disc heights preoperatively and at follow-up.

	Group A	Group B	Group C	Group D	*p* value
Adjacent disc height					
Preoperative	6.9 ± 1.9	6.6 ± 1.8	6.5 ± 1.5	6.4 ± 1.4	0.346
Follow-up	6.1 ± 1.7	6.0 ± 1.8	6.2 ± 1.7	6.0 ± 1.5	0.519
*p* value	<0.001	<0.001	0.079	0.012	
Control disc height					
Preoperative	5.7 ± 1.8	5.7 ± 1.6	6.0 ± 1.9	5.3 ± 1.7	0.496
Follow-up	5.3 ± 1.6	5.4 ± 1.7	5.8 ± 1.7	5.1 ± 1.9	0.218
*p* value	0.089	0.138	0.269	0.301	

## Discussion

The advantages of the VCA include its minimally invasive nature, good biomechanical support, and satisfactory clinical outcomes. Regarding the adverse effects of vertebral augmentation, researchers have mainly focused on intraoperative complications, such as cement extravasation, and the postoperative negative effects of cement injection on adjacent vertebral bodies (6, 19). The influence of vertebral augmentation on adjacent intervertebral discs has rarely been discussed in previous studies. A prospective study of 97 patients conducted by Qian et al. (7) compared the grade of disc degeneration above the fractured vertebra on MR between the PVP *t* and conservative treatment groups. They discovered that the incidence of degeneration of the adjacent intervertebral disc above the fractured vertebra was significantly higher in the vertebral augmentation group than in the control group (52.5% vs. 29.0%). Similar results were indicated in retrospective studies conducted by Pachowsky et al. (20), Lu et al. (8), and Wang et al. (9). However, a long-term follow-up study by König et al. (10) found that vertebral cement augmentation using PKP had no significant effect on disc degeneration. As König's study included discs with preoperative Pfirrmann grade ≥3, the endplate nutrition could have already been affected and could cause bias in evaluating the actual effect of cement augmentation. Li et al. (17) suggested that adjacent disc degeneration might be attributable to the endplate injury of the fractured vertebrae, which was ignored in the above studies. The design defects of the aforementioned studies might be the reason for the discordance in the results. Furthermore, previous studies did not consider one key factor—cement distribution—that might have an impact on adjacent discs by altering both the nutrient supply to the discs and local biomechanics. To determine the effect of bone cement distribution on adjacent intervertebral disc degeneration after vertebral augmentation, we used strict inclusion criteria to reduce potential bias. In our study, we included patients only with upper endplate injuries and the Pfirrmann score of the adjacent discs ≤2. Additionally, endplate injuries are often accompanied by disc injuries, which have been proven to have an important impact on disc degeneration (21) and could therefore influence the evaluation of the actual effect of cement augmentation. Therefore, the influence of disc injuries should be considered. Therefore, patients with severe adjacent disc injury were excluded, and the DDC definition excluded deterioration from grade 1 to grade 2 Pfirrmann scores to further diminish the bias induced by the potentially inaccurate evaluation of the preoperative Pfirrmann score of injured discs on MR.

Several patient characteristics, such as age, sex, body weight, and smoking, have been reported as risk factors for intervertebral disc degeneration (22, 23). In this study, these risk factors were evaluated to improve the reliability of our conclusions. None of these characteristics showed statistically significant difference among the four groups. In the present study, the incidence of degeneration in control discs was not significantly different among the four groups. The number of DDC cases in adjacent discs was significantly higher than that in control discs in groups A, B, and D, but the difference between adjacent and control discs in group C was not statistically significant. These results suggested less severe degenerative changes in the adjacent discs in group C. Further analysis showed a significant difference in the incidence of adjacent disc degeneration among groups A, B, C, and D. Compared with group C, patients in group A had a significantly higher number of DDC cases. Patients in groups B and D also had a higher number of DDC cases than those in group C, although the difference was not statistically significant. These results indicate that vertebral augmentation has an impact on adjacent disc degeneration, which is largely influenced by bone cement distribution. Cement distribution proximal to the injured endplate could accelerate the adjacent disc degeneration, and cement in contact with both the cranial and distal endplates might have a greater adverse impact on adjacent discs. However, cement distributed distally to the injured endplate may have no significant influence on disc degeneration. The alteration in the adjacent disc height further supports this conclusion. The adjacent disc heights decreased significantly at follow-up compared with baseline in groups A, B, and D, whereas the decrease in group C was not statistically significant. However, the control discs, did not exhibit a significant decrease in disc heights. These results were consistent with those of previous reports (9, 17), and further proved the fewer degenerative changes in the adjacent discs in group C. However, the adjacent disc heights at follow-up were not significantly different among the four groups. This might be attributed to the comparatively short follow-up time, as the adjacent disc degeneration was still in the early stages and the decrease in disc height was minor. Similarly, in a previous report with comparable follow-up time (2 years), the height loss of adjacent disc in the vertebral augmentation group was not significantly different from that in the control group (7). Disc height is not strongly related to Pfirrmann scores from grade 1 to 4, and only significantly differentiate between grade 4 and 5 Pfirrmann scores (24, 25). However, no disc degenerated to grade 5 at final follow-up in this study, that might make the disc height change less significant. Significant disc height loss and high-grade disc degeneration were observed in some studies with long-term follow-up (ranging from 8 to 17 years) (26–28). Thus, prolonging the follow-up time may be helpful to observe the differences of disc height change among the four groups.

However, the pathogenesis of disc degeneration remains unclear. Nevertheless, alteration of local biomechanics and impairment of nutrient supply have been suggested to accelerate adjacent disc degeneration after vertebral augmentation according to previous reports. Biomechanical studies have shown significant decreases in adjacent disc pressure after vertebral fractures (29–31). A finite-element study by Luo et al. (29) reported that vertebral injury reduced the intradiscal pressure at the affected level to an average of 47% of the pre-fracture values. These results are consistent with those of previous studies (30, 31). Bone cement augmentation can enhance strain on the intervertebral disc by increasing the elastic modulus of the injured endplate and further altering the local biomechanical profile. A finite element study by Polikeit et al. (32) showed that the maximum pressure increased by 16% above the treated level and by 13% below the treated level. Ananthakrishnan et al. (30) also reported an increased nuclear pressure after augmentation treatment. Similar results have been reported in other finite-element studies (31, 33, 34). Thus, cement distribution closer to the injured cranial endplate in groups A, B, and D may have made the endplates stiffer and induced a higher incidence of adjacent disc degeneration. Bone cement distributed distal to the injured endplate, as shown in group C, would accordingly exert less stress on the adjacent disc and therefore induce less disc degeneration. Additionally, clinical studies have suggested a correlation between disc degeneration and increased adjacent bone density (35, 36). Previous studies also reported that cement distribution close to the endplate is associated with a higher risk of adjacent vertebral fractures (13, 37). Sun et al. (13) reported that cement distribution proximal to the adjacent vertebral body (distributed at the disc level or endplate level) could increase the risk of post- vertebroplasty fractures in adjacent vertebral bodies. Similarly, Zhao et al. (37) found that the diffusion of bone cement into cracks in the injured endplate may increase the incidence of adjacent vertebral fractures. These results further explain the differences in the numbers of DDC cases induced by different cement distribution patterns. Furthermore, bone cement in group A contacted both the cranial and distal endplates could induce to higher elastic modulus as there were no “buffering bones”, which might exert greater stress on peripheral structures and thus induce to greater disc degeneration.

Impairment of the nutrient supply to the disc has been identified as another important factor affecting adjacent disc degeneration. As the largest inert structure, the intervertebral disc is largely dependent on endplate diffusion for nutrient supply (38), and can be impaired by cement augmentation by damaging the capillaries underneath the endplate. Signs of adjacent disc degeneration followed vertebral augmentation were reported (15, 16). However, conclusions from adult animal models (goats, sheep, and dogs) are contradictory (39–41). Because the vertebrae in these animal models were intact, these conclusions may not be applicable to fractured vertebrae. Our study indicated that a combination of vertebral fracture and cement distribution proximal to the injured endplate may be required to influence the nutrient supply and accelerate the degeneration, as the incidence of disc degeneration increased in groups A, B, and D, but not in group C.

This study had several limitations. First, the strict inclusion and exclusion criteria may have led to sampling bias and comparatively small sample sizes in the subgroups. Second, the distribution of bone cement was only evaluated on x-ray films, owing to the lack of postoperative CT images. Third, the severity of endplate injuries was not evaluated due to the lack of a proper rating system, which may have resulted in biased results. Fourth, the average follow-up time was relatively short, which may not have been sufficient to fully evaluate the degenerative condition of the adjacent discs. As no disc degenerated to grade 5 in this study, it might make the disc height change less significant.

## Conclusion

The present study suggests that bone cement distribution influences adjacent disc degeneration after vertebral augmentation in osteoporotic vertebral compression fractures. Cement distribution proximal to the injured endplate can accelerate adjacent disc degeneration, and cement in contact with both the cranial and distal endplates can induce a higher incidence of adjacent disc degeneration.

## Data Availability

The original contributions presented in the study are included in the article/Supplementary Material, further inquiries can be directed to the corresponding author.

## References

[1] Papanastassiou ID Phillips FM Van Meirhaeghe J Berenson JR Andersson GB Chung G Comparing effects of kyphoplasty, vertebroplasty, and non-surgical management in a systematic review of randomized and non-randomized controlled studies. Eur Spine J. (2012) 21(9):1826–43. 10.1007/s00586-012-2314-z 22543412 PMC3459114

[2] Beall D Lorio MP Yun BM Runa MJ Ong KL Warner CB. Review of vertebral augmentation: an updated meta-analysis of the effectiveness. Int J Spine Surg. (2018) 12(3):295–321. 10.14444/5036 30276087 PMC6159665

[3] Mattie R Laimi K Yu S Saltychev M. Comparing percutaneous vertebroplasty and conservative therapy for treating osteoporotic compression fractures in the thoracic and lumbar spine: a systematic review and meta-analysis. J Bone Joint Surg Am. (2016) 98(12):1041–51. 10.2106/jbjs.15.00425 27307365

[4] Clark W Bird P Gonski P Diamond TH Smerdely P McNeil HP Safety and efficacy of vertebroplasty for acute painful osteoporotic fractures (vapour): a multicentre, randomised, double-blind, placebo-controlled trial. Lancet. (2016) 388(10052):1408–16. 10.1016/s0140-6736(16)31341-1 27544377

[5] Trout AT Kallmes DF Kaufmann TJ. New fractures after vertebroplasty: adjacent fractures occur significantly sooner. Am J Neuroradiol. (2006) 27(1):217–23. PMID: 16418388 PMC7976057

[6] Park JS Park YS. Survival analysis and risk factors of new vertebral fracture after vertebroplasty for osteoporotic vertebral compression fracture. Spine J. (2021) 21(8):1355–61. 10.1016/j.spinee.2021.04.022 33971326

[7] Qian J Yang H Jing J Zhao H Ni L Tian D The early stage adjacent disc degeneration after percutaneous vertebroplasty and kyphoplasty in the treatment of osteoporotic vcfs. PLoS One. (2012) 7(10):e46323. 10.1371/journal.pone.0046323 23056283 PMC3466231

[8] Lu X Yang J Zhu Z Lv X Wu J Huang J Changes of the adjacent discs and vertebrae in patients with osteoporotic vertebral compression fractures treated with or without bone cement augmentation. Spine J. (2020) 20(7):1048–55. 10.1016/j.spinee.2020.02.012 32105771

[9] Wang T Si F Zang L Fan N Yuan S Du P Radiographic adjacent segment degeneration and risk factors for osteoporotic vertebral compression fractures treated with percutaneous kyphoplasty. Int Orthop. (2022) 46(11):2619–28. 10.1007/s00264-022-05510-1 35864260

[10] König MA Panzer S Schulz J Bierschneider M Boszczyk BM. Magnetic resonance imaging changes of intervertebral discs after kyphoplasty. Eur Spine J. (2015) 24(4):724–33. 10.1007/s00586-014-3244-8 24664426

[11] Bae JS Park JH Kim KJ Kim HS Jang IT. Analysis of risk factors for secondary new vertebral compression fracture following percutaneous vertebroplasty in patients with osteoporosis. World Neurosurg. (2017) 99:387–94. 10.1016/j.wneu.2016.12.038 28012889

[12] Lee HJ Park J Lee IW Yi JS Kim T. Clinical, radiographic, and morphometric risk factors for adjacent and remote vertebral compression fractures over a minimum follow-up of 4 years after percutaneous vertebroplasty for osteoporotic vertebral compression fractures: novel three-dimensional voxel-based morphometric analysis. World Neurosurg. (2019) 125:e146–e57. 10.1016/j.wneu.2019.01.020 30682507

[13] Sun YC Teng MM Yuan WS Luo CB Chang FC Lirng JF Risk of post-vertebroplasty fracture in adjacent vertebral bodies appears correlated with the morphologic extent of bone cement. J Chin Med Assoc. (2011) 74(8):357–62. 10.1016/j.jcma.2011.06.008 21872816

[14] He X Li H Meng Y Huang Y Hao DJ Wu Q Percutaneous kyphoplasty evaluated by cement volume and distribution: an analysis of clinical data. Pain Physician. (2016) 19(7):495–506. PMID: 27676666

[15] Kang R Li H Ringgaard S Rickers K Sun H Chen M Interference in the endplate nutritional pathway causes intervertebral disc degeneration in an immature porcine model. Int Orthop. (2014) 38(5):1011–7. 10.1007/s00264-014-2319-9 24652423 PMC3997759

[16] Feng Z Chen L Hu X Yang G Chen Z Wang Y. Vertebral augmentation can induce early signs of degeneration in the adjacent intervertebral disc: evidence from a rabbit model. Spine. (2018) 43(20):E1195–e203. 10.1097/brs.0000000000002666 29649084

[17] Li Y Su Q Feng X Li L Tan J Ke R. The role of endplate injury in intervertebral disc degeneration after vertebral augmentation in ovcf patients. Front Surg. (2022) 9:1091717. 10.3389/fsurg.2022.1091717 36704508 PMC9871805

[18] Pfirrmann CW Metzdorf A Zanetti M Hodler J Boos N. Magnetic resonance classification of lumbar intervertebral disc degeneration. Spine. (2001) 26(17):1873–8. 10.1097/00007632-200109010-00011 11568697

[19] Yeom JS Kim WJ Choy WS Lee CK Chang BS Kang JW. Leakage of cement in percutaneous transpedicular vertebroplasty for painful osteoporotic compression fractures. J Bone Joint Surg Br. (2003) 85(1):83–9. 10.1302/0301-620x.85b1.13026 12585583

[20] Pachowsky ML Kleyer A Wegener L Langenbach A Simon D Janka R Quantitative T2 mapping shows increased degeneration in adjacent intervertebral discs following kyphoplasty. Cartilage. (2020) 11(2):152–9. 10.1177/1947603518758434 29553284 PMC7097981

[21] Alkhatib B Rosenzweig DH Krock E Roughley PJ Beckman L Steffen T Acute mechanical injury of the human intervertebral disc: link to degeneration and pain. Eur Cell Mater. (2014) 28:98–110; discussion-1. 10.22203/ecm.v028a08 25214017

[22] Teraguchi M Yoshimura N Hashizume H Yamada H Oka H Minamide A Progression, incidence, and risk factors for intervertebral disc degeneration in a longitudinal population-based cohort: the Wakayama spine study. Osteoarthritis Cartilage. (2017) 25(7):1122–31. 10.1016/j.joca.2017.01.001 28089899

[23] Kirnaz S Capadona C Wong T Goldberg JL Medary B Sommer F Fundamentals of intervertebral disc degeneration. World Neurosurg. (2022) 157:264–73. 10.1016/j.wneu.2021.09.066 34929784

[24] Salamat S Hutchings J Kwong C Magnussen J Hancock MJ. The relationship between quantitative measures of disc height and disc signal intensity with pfirrmann score of disc degeneration. Springerplus. (2016) 5(1):829. 10.1186/s40064-016-2542-5 27386278 PMC4917505

[25] Emanuel KS Kingma I Helder MN Smit TH. Response to: ‘A dose-response relationship between severity of disc degeneration and intervertebral disc height in the lumbosacral spine’. Arthritis Res Ther. (2016) 18:41. 10.1186/s13075-016-0944-y 26852747 PMC4745160

[26] Videbaek TS Egund N Christensen FB Grethe Jurik A Bünger CE. Adjacent segment degeneration after lumbar spinal fusion: the impact of anterior column support: a randomized clinical trial with an eight- to thirteen-year magnetic resonance imaging follow-up. Spine. (2010) 35(22):1955–64. 10.1097/BRS.0b013e3181e57269 20959776

[27] Ekman P Möller H Shalabi A Yu YX Hedlund R. A prospective randomised study on the long-term effect of lumbar fusion on adjacent disc degeneration. Eur Spine J. (2009) 18(8):1175–86. 10.1007/s00586-009-0947-3 19337757 PMC2899511

[28] Schulte TL Leistra F Bullmann V Osada N Vieth V Marquardt B Disc height reduction in adjacent segments and clinical outcome 10 years after lumbar 360 degrees fusion. Eur Spine J. (2007) 16(12):2152–8. 10.1007/s00586-007-0515-7 17922149 PMC2140131

[29] Luo J Annesley-Williams DJ Adams MA Dolan P. How are adjacent spinal levels affected by vertebral fracture and by vertebroplasty? A biomechanical study on cadaveric spines. Spine J. (2017) 17(6):863–74. 10.1016/j.spinee.2017.01.013 28167249

[30] Ananthakrishnan D Berven S Deviren V Cheng K Lotz JC Xu Z The effect on anterior column loading due to different vertebral augmentation techniques. Clin Biomech. (2005) 20(1):25–31. 10.1016/j.clinbiomech.2004.09.004 15567533

[31] Luo J Skrzypiec DM Pollintine P Adams MA Annesley-Williams DJ Dolan P. Mechanical efficacy of vertebroplasty: influence of cement type, bmd, fracture severity, and disc degeneration. Bone. (2007) 40(4):1110–9. 10.1016/j.bone.2006.11.021 17229596

[32] Polikeit A Nolte LP Ferguson SJ. The effect of cement augmentation on the load transfer in an osteoporotic functional spinal unit: finite-element analysis. Spine. (2003) 28(10):991–6. 10.1097/01.Brs.0000061987.71624.17 12768136

[33] Keller TS Kosmopoulos V Lieberman IH. Vertebroplasty and kyphoplasty affect vertebral motion segment stiffness and stress distributions: a microstructural finite-element study. Spine. (2005) 30(11):1258–65. 10.1097/01.brs.0000163882.27413.01 15928549

[34] Baroud G Bohner M. Biomechanical impact of vertebroplasty. Postoperative biomechanics of vertebroplasty. Joint Bone Spine. (2006) 73(2):144–50. 10.1016/j.jbspin.2005.02.004 16095945

[35] Wang Y Boyd SK Battié MC Yasui Y Videman T. Is greater lumbar vertebral bmd associated with more disk degeneration? A study using Μct and discography. J Bone Miner Res. (2011) 26(11):2785–91. 10.1002/jbmr.476 21786320

[36] Pye SR Reid DM Adams JE Silman AJ O'Neill TW. Radiographic features of lumbar disc degeneration and bone mineral density in men and women. Ann Rheum Dis. (2006) 65(2):234–8. 10.1136/ard.2005.038224 16014671 PMC1798008

[37] Zhao Z Deng L Hua X Liu H Zhang H Jia X A retrospective study on the efficacy and safety of bone cement in the treatment of endplate fractures. Front Surg. (2022) 9:999406. 10.3389/fsurg.2022.999406 36277290 PMC9585934

[38] Fields AJ Ballatori A Liebenberg EC Lotz JC. Contribution of the endplates to disc degeneration. Curr Mol Biol Rep. (2018) 4(4):151–60. 10.1007/s40610-018-0105-y 30546999 PMC6287619

[39] Krebs J Ferguson SJ Goss BG Stauffer E Ettinger L Aebli N. Effect of vertebral cement augmentation with polymethylmethacrylate on intervertebral disc and bone tissue. J Biomed Mater Res B Appl Biomater. (2012) 100(3):660–7. 10.1002/jbm.b.31990 22121034

[40] Hutton WC Murakami H Li J Elmer WA Yoon ST Minamide A The effect of blocking a nutritional pathway to the intervertebral disc in the dog model. J Spinal Disord Tech. (2004) 17(1):53–63. 10.1097/00024720-200402000-00011 14734977

[41] Verlaan JJ Oner FC Slootweg PJ Verbout AJ Dhert WJ. Histologic changes after vertebroplasty. J Bone Joint Surg Br. (2004) 86(6):1230–8. 10.2106/00004623-200406000-00016 15173297

